# EEG Transients in the Sigma Range During non-REM Sleep Predict Learning in Dogs

**DOI:** 10.1038/s41598-017-13278-3

**Published:** 2017-10-11

**Authors:** Ivaylo Borislavov Iotchev, Anna Kis, Róbert Bódizs, Gilles van Luijtelaar, Enikő Kubinyi

**Affiliations:** 1 Department of Ethology, Eötvös Loránd University, Budapest, Hungary; 2 Institute of Cognitive Neuroscience and Psychology, Hungarian Academy of Sciences, Budapest, Hungary; 3 Institute of Behavioural Sciences, Semmelweis University, Budapest, Hungary; 4 Donders Centre of Cognition, Radboud University, Nijmegen, The Netherlands

## Abstract

Sleep spindles are phasic bursts of thalamo-cortical activity, visible in the cortex as transient oscillations in the sigma range (usually defined in humans as 12–14 or 9–16 Hz). They have been associated with sleep-dependent memory consolidation and sleep stability in humans and rodents. Occurrence, frequency, amplitude and duration of sleep spindles co-vary with age, sex and psychiatric conditions. Spindle analogue activity in dogs has been qualitatively described, but never quantified and related to function. In the present study we used an adjusted version of a detection method previously validated in children to test whether detections in the dogs show equivalent functional correlates as described in the human literature. We found that the density of EEG transients in the 9–16 Hz range during non-REM sleep relates to memory and is characterized by sexual dimorphism similarly as in humans. The number of transients/minute was larger in the learning condition and for female dogs, and correlated with the increase of performance during recall. It can be concluded that in dogs, automatic detections in the 9–16 Hz range, in particular the slow variant (<13 Hz), are functional analogues of human spindles.

## Introduction

Studies relying on electroencephalography (EEG) to study the canine brain have so far focused on large-scale neural dysfunctions like epilepsy^1,2^ or oscillations signalling levels of attention, vigilance or sleep^3–5^. Yet in order to refine the level of detail and to extract more information, research needs to focus on EEG transients – short latency signatures of functional events – such as sleep spindles.

Sleep spindles are brief episodes of brain activity, at least 0.5 seconds long^6,7^, observed during mammalian non-REM sleep^8,9^ and can be measured on the scalp surface using EEG. They are described as symmetrical around the baseline, monomorphic and biphasic waves^8^ which typically show a rise and subsequent decline in amplitude. Strong inter-individual variation has been described for both amplitude and frequency^10^. These parameters are, however, stable within individuals^11^ and normally distributed^12^. Although some detection methods have tried to integrate more complex criteria^13,14^, many findings associated with functional correlates rely on a relatively simple frequency-amplitude-duration definition^15–17^. The proposed values for the frequency of a human spindle vary between strict 12–14 Hz boundaries^6^ and more inclusive 9–16 Hz^18^ or 11–16 Hz^16^ definitions, also referred to as the “sigma band”. Some authors also distinguish slow and fast spindles^18–20^. Slow spindles are found predominantly in frontal derivations; they exhibit a frequency of 10.25–12 Hz^20^, close to the frequency of the alpha band^21^. Alpha activity is most clearly observed during relaxed wakefulness. Fast spindles, more commonly observed in central and posterior derivations, have an average cycle of 14 Hz and can reach up to 16 Hz^18,19,21^.

Sleep spindles are an ideal target to advance the scope of non-invasive investigations into the dog brain via surface EEG due to established functional correlates and knowledge of the underlying mechanisms. The thalamo-cortical circuit underlying spindle activity^22,23^ is thought to block bottom-up information transfer from subcortical areas and the hippocampus by rendering the cortex unresponsive^24,25^. Spindles have been therefore hypothesized to promote memory consolidation by suppressing conflicting information^26^ that could interfere with cortico-cortical encoding. Accordingly, the density (occurrence per minute non-REM sleep) and absolute number of spindles were found to predict overnight memory consolidation in humans^16,27,28^ and rats^29,30^. It has also been shown that interfering with spindle activity using Transcranial Alternating Current (TAC) affects motor learning^15^, and that spindles are predicted by hippocampal ripples^31,32^, which are linked to replay of spatial information during sleep^33^ and wakefulness^34^. The gating function of spindles has also been reasoned to underlie sleep stability^35^ and spindle density predicts sleep stability during exposure to noise^36^. Memory and sleep stability decline with age^37,38^ and so do the density, amplitude and duration of sleep spindles^39–41^, particularly in Alzheimer dementia^42^. Some studies suggest a functional difference between fast and slow spindles. Increased fast spindle activity was shown to aid the learning of motor sequences^15^, whereas frontal (presumably slow) spindles were associated with verbal recall^16^. In addition, changes and differences in the expression of sleep spindles and their features are reported as the result of sexual dimorphism, intelligence and various psychiatric conditions: amplitudes, duration and occurrence are higher than usual in depression^43^, while overall reduced in schizophrenia^44,45^. Women on average express a higher number of spindles^46,47^ and women’s IQ scores correlate with higher fast spindle amplitudes^48–50^.

So far sleep spindles have only been described and never quantified in the dog. One assertion is that they are less salient than in human recordings^3,51^. Attempts to estimate their frequency vary strongly, including 2–5 Hz^9^, 5–12 Hz^1^ and 12–16 Hz^3^. The low overlap in these suggestions is a sign of poor inter-rater reliability, which poses the question if visual inspection can be used to validate detection methods as is recommended in humans^52^. Meanwhile, automated algorithms operate on the assumption that spindles need to be recovered from a superimposition of oscillations, which will at times render them invisible even for experts^13,14,16,17^.

We therefore decided to evaluate three spindle detection methods in the dog based on the reasoning that an analogy between human and dog spindles is more warranted if it can be related to the same function in both species. To this end we tested how well a fully automated search can reproduce relationships described in humans and rodents. We designed a Matlab detection script applying the criteria proposed by Nonclercq *et al*.^53^ which combines a frequency(power)-amplitude-duration approach with additional outlier-control (normal modelling). Our version of the algorithm was tested with three different frequency definitions, corresponding to the strict 12–14 Hz range^6,53^, the broader 9–16 Hz range^18^ and a 5–12 Hz range proposed specifically for dogs by Pákozdy *et al*.^1^. The latter was derived from an explorative, visual inspection of the EEG transients. The data set that we used was from Kis *et al*.^4^ from an experiment specifically designed to test the contributions of sleep to memory consolidation.

## Methods

### Ethics statement

Research was carried out in accordance with the Hungarian regulations on animal experimentation and the Guidelines for the use of animals in research described by the Association for the Study Animal Behaviour (ASAB). The Hungarian “Animal Experiments Scientific and Ethical Committee” issued a statement (under the number PE/EA/853–2/2016), approving our experimental protocol by categorizing it as a non-invasive study that causes less pain or suffering than the equivalent of inserting a needle. All owners volunteered to participate in the study.

### Subjects and Behavioural paradigm (adapted from Kis *et al*.^4^

**Figure 1:**
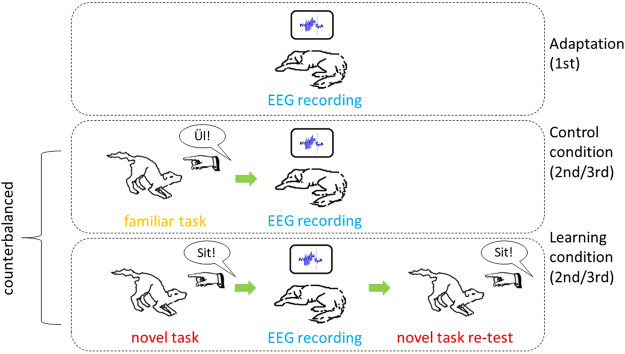
All dogs first attended an adaptation session and polysomnographic data was collected for three hours. Subsequently the learning and control condition followed in random order, each including another three hours recording session. Illustrations by first author.

15 adult pet dogs, mean age ± SD: 3.67 ± 1.91; 8 males, 7 females; from 7 pure breeds (3 Border Collies, 2 Golden Retrievers, 1 Labrador Retriever, 1 Poodle, 1 Belgian Shepherd, 1 Puli, 1 Miniature Schnauzer) and 3 mixed breeds (3 unknown, 1 mixed Briard and 1 mixed Malinois), participated three times in 3-hour-long polysomnography recordings^3^, on a total of 3 days (see Fig. 1).

### Protocol

The first session was an adaptation, followed by a control (non-learning) and learning session in a counterbalanced order (a total of three experimental sessions). The dogs had three hours’ time to sleep on each of these occasions and the EEG data were obtained from these sleeping periods.

7 out of 15 dogs (46.7%, group I) started with the control (non-learning) condition first. Dogs of group II received the reversed order. The interval between the two sessions varied from 1 to 38 days, 10.5 ± 2.9 (mean ± SE) with no difference between the groups.

In the learning condition dogs were taught to perform two familiar actions (sit and lie down), using unfamiliar commands (English phrases instead of Hungarian, the language in which the dogs were originally trained). The training procedure followed a standardized schedule concluding with an 18-trial baseline test (for details see Supplements in Kis *et al*.^4^). In the non-learning condition the dogs had to execute an identical sequence of “Sit!” and “Lie down!” actions, but this time the experimenter used the familiar commands, accompanied by familiar hand signals. The learning and non-learning tasks were followed by a 3-hour-long polysomnography recording each. In the learning condition, the polysomnography recording was followed by another 18-trial session where dogs had to execute the previously learned English commands (Retest). Performance was measured in each condition as the percentage of correctly followed commands (but see Supplementary Table S1 for the absolute number correct responses in each session). For the present work the variable’learning gain’ was used for comparisons within the learning condition alone, calculated as the difference of performance scores before and after sleep.

### Polysomnographic method

The specifics of the polysomnographic method are detailed in previous publications^3,4^. The signal for our EEG analyses comes from a frontal electrode placed on the anterior of the skull midline (Fz) which was corrected (differential recording) for activity from a second recording electrode placed centrally along the same axis (Fz-Cz). In addition, the setting included also a ground electrode at the left musculus temporalis. The impedance of the active electrodes was kept below 15 kΩ and the signal was collected, pre-filtered, amplified and digitized with a sampling rate of 249 Hz by using a 30-channel Flat Style SLEEP La Mont Headbox with implemented second order filters (high pass above 0.5 Hz and low pass below 70 Hz) as well as a HBX32-SLP 32 channel pre-amplifier (La Mont Medical Inc., USA). The EEG signal was divided in sleep stages during the original study using visual scoring^4^.

### Detection algorithm

All steps of the algorithm were implemented in Matlab^®^. Detections were obtained from parts of the EEG marked as non-REM sleep in Kis *et al*.^4^ (the individual stages of non-REM sleep are not clearly distinguishable in the dog). The EEG signal (Fz-Cz) was filtered to remove electrical noise and artefacts (maximal frequency of stop band 3 Hz, high pass 5 Hz; low pass 16 Hz, minimal frequency of stop band 35 Hz using a Butterworth filter with less than 0.5 dB ripple in the pass band and 30 dB attenuation), then analysed with a Fast Fourier Transform (FFT) of 125 ms overlapping, Hanning-tapered 500 ms windows and zero-padded to support a 0.1 Hz resolution. The window length corresponds to the minimum duration of a spindle^6^ and is in accordance with previous propositions for moving windows^7,53^ targeting spindle detection. Except for the pre-filtering, all the above steps are adopted from Nonclercq *et al*.^53^. Because canine EEG recordings are more noisy^3,4^ and obtained by a single bipolar derivation^3^, our filter settings excluded more frequencies than proposed in the original description of the method^53^ in order to ensure a higher signal to noise ratio. In addition, we also repeated each search with one harmonic below and above the target frequency to ensure any effect discovered with that target was not due to random noise or spectrally similar, sharp-wave, epileptoform activity. Three automated criteria were then used to determine if a time-window was occupied by a spindle:

1. The maximum peak power of a given segment was within the target range^53^ (12–14 Hz^6^, 9–16 Hz^18^, 5–12 Hz^1^ respectively).
2. Amplitudes were calculated for each window as the root mean square (rms) of the corresponding signal segment^53^. The amplitude of a spindle was previously proposed to be one standard deviation from the amplitude of the baseline^6,53^, which was the filtered total of non-REM epochs^53^. Because the EEG traces displayed high inter-individual variation in amplitude, standard scores (Z) were calculated for each window’s rms value relative to the population of rms values for that dog and session. If the standard score for a window was ≥ 1 the event was considered large enough to be part of spindle activity. For the control search based on the harmonics of the target frequencies, the segment was chosen twice as long for the first lower harmonic and half as long for the first higher harmonic.
3. In humans, spindles were previously found to follow a normal distribution in both amplitude and frequency within individuals^12,53^. To account for each subject’s individual range on these measures, for both amplitude and frequency the true means and standard deviations of an individual dog and session were estimated using a maximum likelihood estimation on the sample of detections obtained with step 1 and 2. This was followed by an altered repetition of the first two criteria: The frequency boundaries of the first criterion were redefined as ±2 standard deviations from the estimated mean and the same was done for amplitude based on the amplitude’s estimated mean and standard deviation (using the standard scores from the 2^nd^ criterion). Due to the latter adjustments the final group of detections could include events with frequencies outside the initial search range, while events outside the adjusted range were discarded as outliers. The events were required to also fulfil the original 2nd criterion with rms standard scores being above 1.

**Figure 2:**
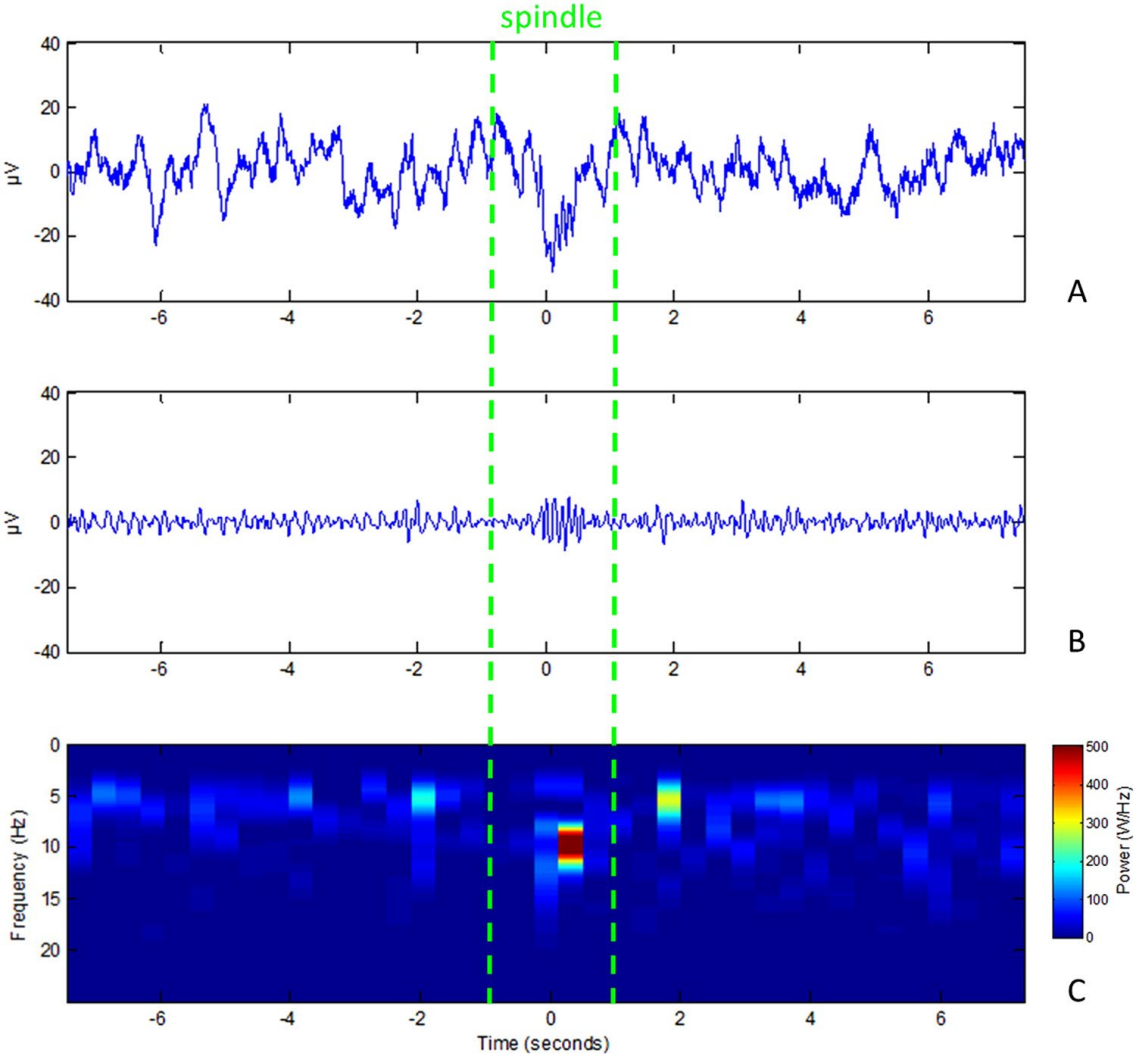
Example event the script marked as a spindle for the 9–16 Hz target band. Timepoint zero marks the centre of a detection and 7.5 seconds of the signal before and after are plotted on a scale from 40 to −40 µV for the raw trace (**A**) and filtered trace (**B**). A time-frequency plot (**C**) displays the change in frequency power for the segment shown in (**A**) and (**B**). The example is taken from the control condition (Maya, 6 year old Golden Retriever ♂).

The time-windows whose content passed these three criteria and two consecutive selections, were used to select from a corresponding array of time-points referencing the centre of each time-window. To automatically estimate the number of separate spindles, the selected time-points that were less than half a second apart^53^ were grouped as referring to the same spindle-event. The average intra-spindle frequency was, however, calculated across all time-windows containing spindle-activity. Amplitudes were measured as the relative distance to baseline, using the standard scores calculated for criterion 2. The time-points were also collected by our algorithm and used to visualize the detections in 15-second-long windows (Fig. 2).

### Statistical Analyses

A generalized linear mixed model (GLMM) was used for comparisons involving more than one predictor. The model used a robust estimation and a Satterthwaite approximation for the degrees of freedom, which is recommended for small sample sizes (N = 15, unless otherwise stated). A Gamma distribution assumption was chosen for most readout variables, since negative values cannot be scored for frequency, amplitude or density (Gamma is recommended for distributions with all positive values). One exception was learning gain (one dog performed worse after sleep) and for this variable the default linear assumption was used, after normality was visually confirmed. Significant effects were further analysed post-hoc, either using the build-in post hoc tests of the GLMM (for categorical variables) or running the model again with a single predictor. Paired-sample t-tests were used for comparisons between conditions. Results were plotted if significant in post-hoc testing (GraphPad Prism).

## Results

For a descriptive exploration of the characteristics of sleep spindles we used the data from the adaptation sessions. Three different criteria for spindles were explored, see Table 1.

**Table 1:** Means and standard errors for the absolute number of detections, occurrence per minute non-REM sleep, their average frequency, and amplitude, shown for each target range tested. On average non-REM sleep in the adaptation session lasted 32.1 ± 7.4 minutes (N = 12, 3 dogs did not sleep and were excluded for calculating the average frequency and amplitude).

search range	detections	detections/minute	frequency (Hz)	amplitude (z-score)
12–14 Hz	8.7 ± 3.02	0.4 ± 0.1	12.9 ± 0.1	1.6 ± 0.1
9–16 Hz	71.1 ± 18.4	3.1 ± 0.4	9.9 ± 0.3	1.9 ± 0.2
5–12 Hz	202.7 ± 51.8	8.6 ± 1.1	6.2 ± 0.1	1.8 ± 0.1

### Age, sex, and learning gain

**Figure 3:**
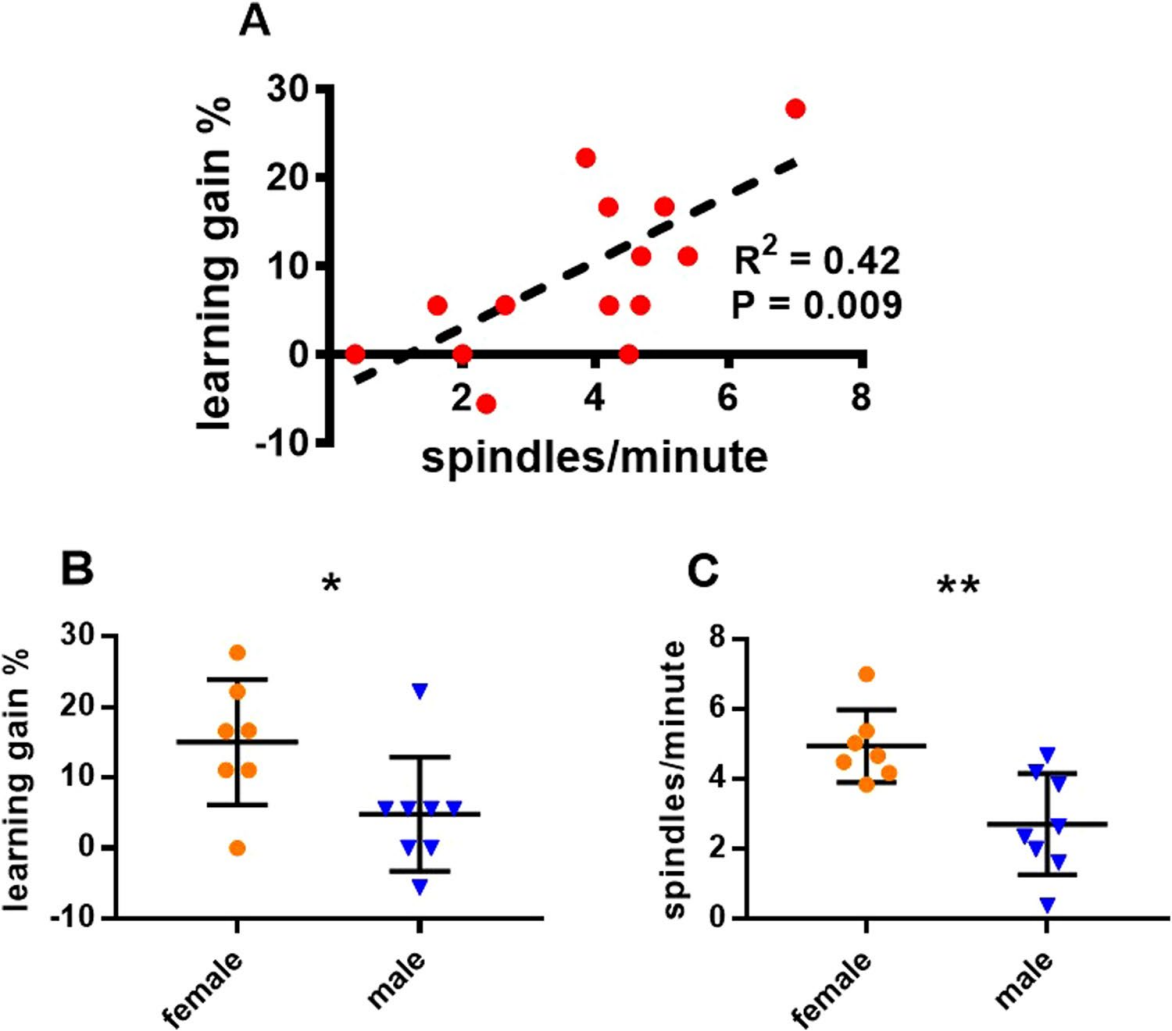
Effects of Learning and Sex on spindle density in the learning condition. Detections in the 9–16 Hz range. (**A**) A scatter plot for the correlation of spindle density (spindles/minute) with learning gain (% increase in performance after sleep). (**B**) Scatter plot, with medians and standard errors of learning gain, comparing female and male dogs (N = 15, 7 ♀, P = 0.022). (**C**) Scatter plot, with medians and standard errors of spindle density in the learning condition, comparing female and male dogs, using an independent samples t-test (N = 15, 7 ♀, P = 0.001)

An initial exploration into how learning gain (difference in percentage correct responses after sleep – before sleep in the learning condition) was predicted by sex and age revealed no effect of age (GLMM, F_1,12_ = 0.759, P = 0.401), but a significant effect of sex (GLMM, F_1,12_ = 6.948, P = 0.022). Females displayed a higher learning gain (15.6 ± 3.6 versus 4.4 ± 2.2, means ± SE, t_12_ = 2.636, P = 0.022), see Fig. 3B.

Next the overall predictive strength of detections from each frequency-definition was compared by testing how age, sex and learning gain would predict spindle density in the learning condition. Transients in the 5–12 Hz and 12–14 Hz range showed no relationship to learning or age (see supplementary). Below we present the results for transients in the 9–16 Hz range (Fig. 3).

We found that spindle density in the learning condition increased with learning gain (GLMM, F_1,11_ = 7.656, P = 0.018). This relationship remained significant in post-hoc testing (GLMM, F_1,13_ = 9.293, P = 0.009, Fig. 3A). Spindle density also increased with age (GLMM, F_1,11_ = 6.492, P = 0.027) and was different for the sexes (GLMM, F_1,11 = _14.489, P = 0.003). Females had a higher spindle density than males (4.75 ± 0.2 versus 2.69 ± 0.4, means ± SE, t_11_ = 4.787, P = 0.001, Fig. 3C), but the effect of age was not significant post-hoc (GLMM, F_1,13_ = 0.178, P = 0.68).

**Figure 4:**
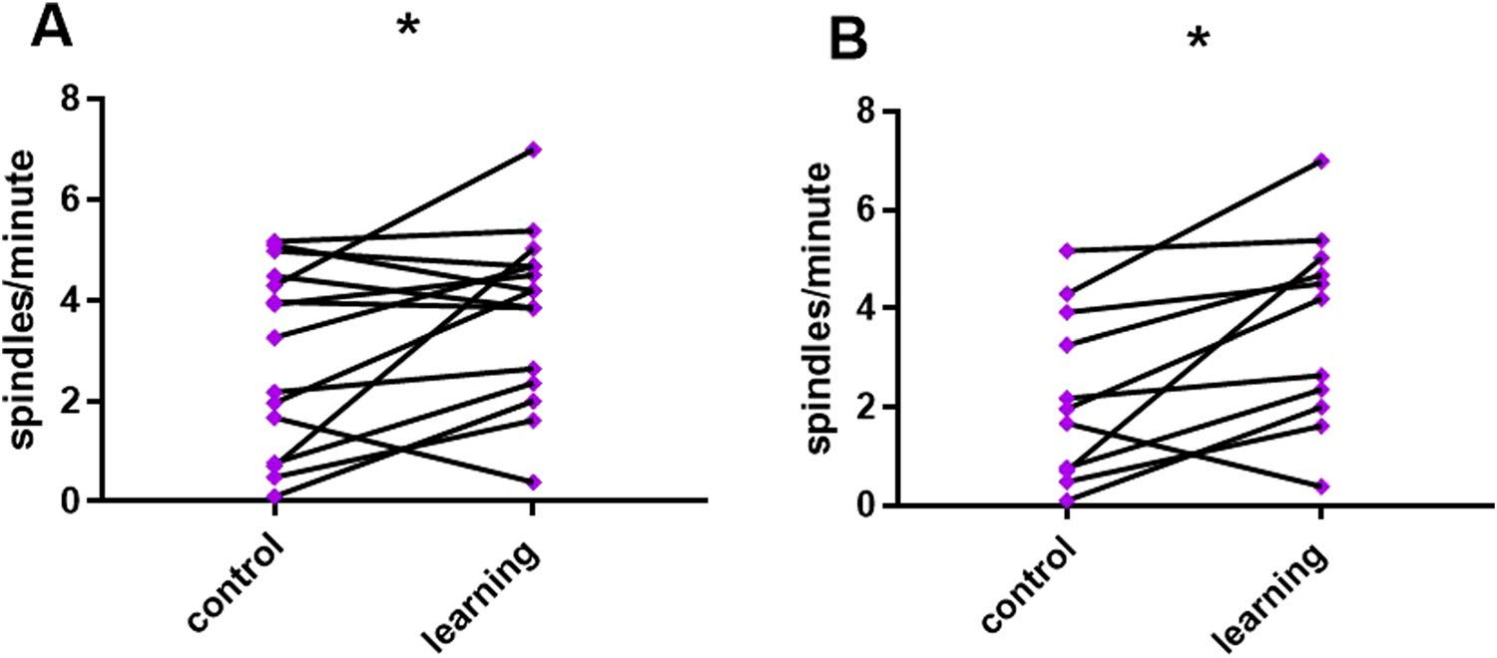
Differences in Density between conditions. Detections in the 9–16 Hz range. (**A**) Before-after plot of spindle density for the learning and control conditions, compared with a paired samples t-test (N = 15, P = 0.04). (**B**) Before-after plot of spindle density for the learning and control conditions, excluding dogs with more than 10 days between sessions, compared with a paired samples t-test (N = 11, P = 0.01).

The difference in density between conditions was tested using paired-sample t-tests. For detections in the 12–14 Hz range the difference was non-significant (t_14_ = 1.002, P = 0.333) and detections in the 5–12 Hz likewise did not display a different density for learning versus control (t_14_ = 1.139, P = 0.274). We found a significant increase in density in the learning condition as compared to the non-learning condition for detections in the 9–16 Hz range (3.76 ± 0.43 versus 2.87 ± 0.47, means ± SE, t_14_ = 2.264 P = 0.04, Fig. 4A). To account for the distance between sessions, we repeated the density comparison for dogs with less than 10 days between the two sessions (N = 11). The difference in density remained significant (3.62 ± 0.59 versus 2.23 ± 0.52, means ± SE, t_10_ = 3.114 P = 0.01, Fig. 4B).

**Figure 5:**
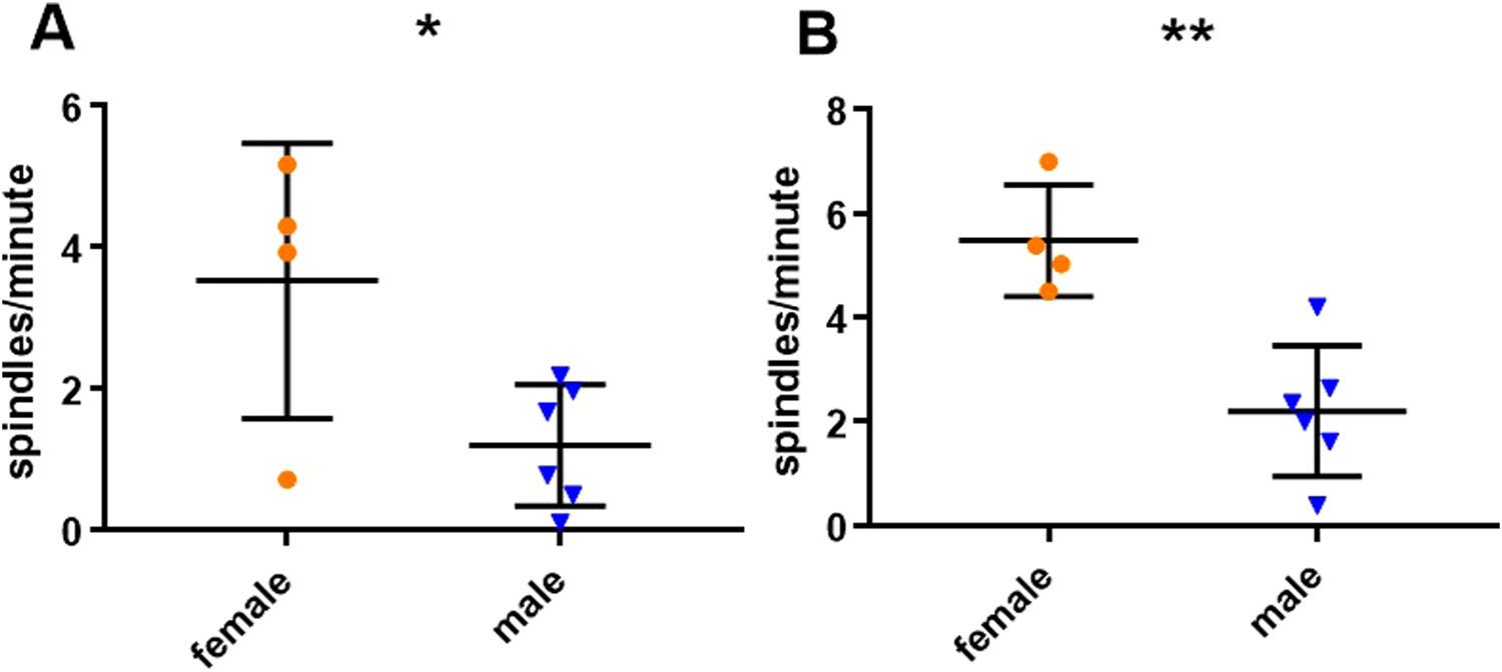
Sex differences (excluding neutered dogs). Detections in the 9–16 Hz range. (**A**) Scatter plot, medians and standard errors of spindle density in the control condition, comparing female and male dogs, using an independent samples t-test (N = 10, 4 ♀, P = 0.03). (**B**) Scatter plot, medians and standard errors of spindle density in the learning condition, comparing female and male dogs, using an independent samples t-test (N = 10, 4 ♀, P = 0.003).

To examine if the effect of sex was dependent on an intact reproductive system we used independent samples t-tests in both conditions, excluding neutered animals (N = 10, 4 ♀). While the effect was visible for all frequency definitions we concentrated on detections in the 9–16 Hz range due to their significant association with learning within and between conditions. Females displayed a significantly higher density of spindles in the control condition (3.5 ± 1 versus 1.2 ± 0.4, means ± SE, t_8_ = 2.627, P = 0.03, Fig. 5A) as well as the learning condition (5.5 ± 0.5 versus 2.2 ± 0.5, means ± SE, t_8_ = 4.259, P = 0.003, Fig. 5B).

### Slow and fast spindles

The next set of tests aimed to inquire if a distinction between fast and slow spindles, now a standard in the human literature^21^, would yield meaningful information in the dog. To this end we focused on the 9–16 Hz range which is deemed to encompass both subtypes^18^. Using the 13 Hz mark (see Schabus *et al*.^54^), we divided the previously obtained 9–16 Hz transients in slow spindles (< = 13 Hz) and fast spindles (> = 13 Hz). The GLMM for effects of learning gain, age and sex in the learning condition as well as the paired samples t-tests of spindle density between conditions was repeated to establish which variety was more specifically connected to learning. In addition, we averaged the amplitudes, frequencies and densities across both conditions and used a GLMM to see if they could be used to predict age, excluding dogs with no detections (3 dogs had no fast spindles, N = 12 for the fast spindles analysis). Because the frequency and amplitude of slow and fast spindles could vary independently as the literature suggests^21,48^ we did not investigate these features for detections in the complete 9–16 Hz range.

1. Slow spindles: In the learning condition the density of slow spindles was significantly predicted by learning gain (GLMM, F_1,11_ = 10.412, P = 0.008). This effect was also significant post-hoc (GLMM, F_1,13_ = 11.661, P = 0.005, Fig. 6A). Sex was a significant predictor (GLMM, F_1,11_ = 7.364, P = 0.02). Females had more spindles/minute than males (4.1 ± 0.3 versus 2.6 ± 0.4, means ± SE, t_11_ = 3.031, P = 0.011, Fig. 6B). There was a trend for density to increase with age (GLMM, F_1,11_ = 4.124, P = 0.067). There was also a trend for more spindles/minute in the learning condition as compared to the control condition (3.4 ± 0.4 versus 2.6 ± 0.5, means ± SE, t_14_ = 2.135, P = 0.051). This effect was significant upon excluding dogs with more than 10 days waiting time between the EEG sessions (3.2 ± 0.5 versus 2.01 ± 0.5, means ± SE, t_10_ = 2.959, P = 0.014, Fig. 6C). Age was not predicted by the mean amplitude (GLMM, F_1,11_ = 0.285, P = 0.604), mean frequency (GLMM, F_1,11_ = 1.351, P = 0.27) or mean density of slow spindles (GLMM, F_1,11_ = 0.673, P = 0.429).

**Figure 6:**
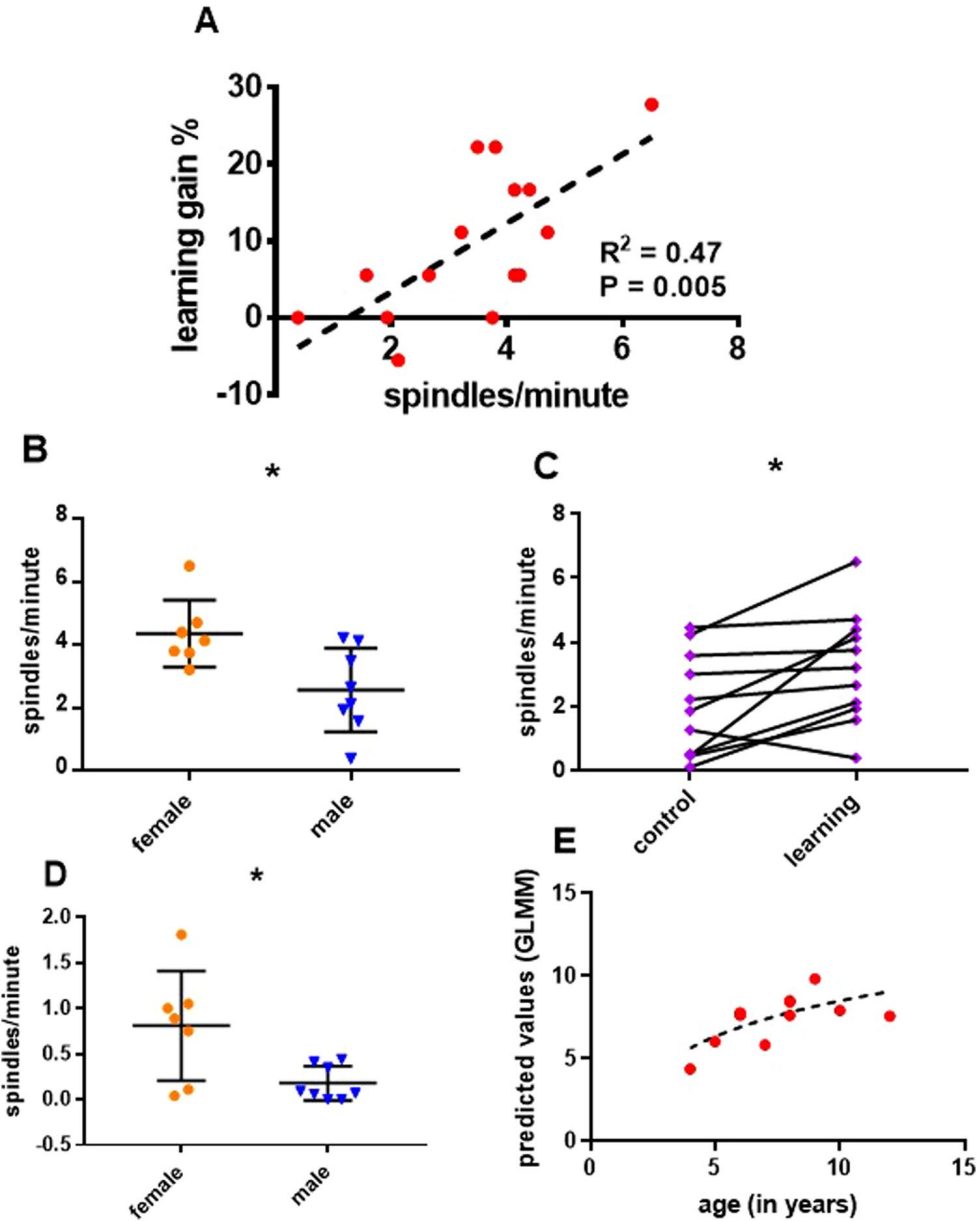
Slow and fast spindles. (**A**) A scatter plot for the correlation of slow spindle density (spindles/minute) with learning gain (% increase in performance after sleep). (**B**) Scatter plot, medians and standard errors of slow spindle density in the learning condition, comparing female and male dogs, using an independent samples t-test (N = 15, 7 ♀, N = 0.011). (**C**) Before-after plot of slow spindle density for the learning and control conditions, excluding dogs with more than 10 days between sessions, compared with a paired samples t-test (N = 11, P = 0.014). (**D**) Scatter plot, medians and standard errors of fast spindle density in the learning condition, comparing female and male dogs, using an independent samples t-test (N = 15, 7 ♀, P = 0.027). (**E**) A scatter plot showing the match between age (in years) and age values predicted by estimates obtained from the mean amplitude, mean frequency and mean density of fast spindles. Dogs without detections were excluded to not skew the estimation of amplitude and frequency (N = 12).
2. Fast spindles: The density of fast spindles was not predicted by learning gain (GLMM, F_1,9_ = 0.005, P = 0.946) or age (GLMM, F_1,9_ = 0.093, P = 0.768) but was significantly predicted by sex (GLMM, F_1,9_ = 10.83, P = 0.009). Females displayed more fast spindles/minute than males (0.8 ± 0.2 versus 0.2 ± 0.1, means ± SE, t_9_ = 2.631, P = 0.027, Fig. 6D). Condition had no effect on the density of fast spindles (t_14_ = 1.557, P = 0.142). Age was predicted by the mean amplitude of fast spindles (GLMM, F_1,8_ = 27.608, P = 0.001), which decreased with years of age; was predicted by a decrease in their density (GLMM, F_1,8_ = 6.454, P = 0.035), while frequency showed a trend to increase with age (GLMM, F_1,8_ = 4.132, P = 0.077). The effect of density was not significant post-hoc (GLMM, F_1,10_ = 0.607, P = 0.454), neither the effect of frequency (GLMM, F_1,10_ = 0.082, P = 0.781), but the mean amplitude remained a significant predictor of age (1.8 ± 0.1 versus 1.5 ± 0.3, means ± SE for dogs older and younger than 6 years^55^, amplitude measured as standard deviation above baseline; GLMM, F_1,10_ = 12.426, P = 0.005). See Fig. 6 for a summary of these results.

## Discussion

The main goal of the present study was to identify, in the dog, functional equivalents of sleep spindles, which are shown by previous work to be promising indicators of thalamo-cortical interactions^23,56,57^ and a marker of sleep-dependent memory consolidation^16,27,29,58^ and aging^39–42^ in humans. Contrary to previous studies, which mostly avoided systematic quantification in the dog and relied solely on visual inspection^1,59^, three variants of previously established automated search criteria^53^ were used.

Although visual inspection was once the golden standard and some authors still advocate using it as an evaluation criterion for automated algorithms^52^, there are several reasons to consider a different approach. The literature suggests that there is little agreement between observers regarding the definition of canine spindling. Pákozdy and colleagues suggest 5–12 Hz^1^, but estimates as low as 2–5 Hz have also been proposed^9^. Some more recent algorithms operate on the assumption that spindles will occasionally be completely hidden by other oscillations and require reconstruction^13,14^. Feedback-coupled online detection has also shown that spindle activity and function can be manipulated successfully without the possibility to double-check the detections visually prior to the intervention^15,60^. An EEG signature that varies strongly across species with regard to a defining feature, like the frequency of a spindle or an epileptoform spike-wave discharge, can none the less have a similar function as it models the corresponding human oscillation^9,61^. Since the dog as a model of human social behavior^62,63^ and (neuro-)cognition^3,64,65^ is a relevant subject for comparative research, the necessity to establish functional analogies has a priority. Therefore, we “validated” our definition of sleep spindles based on analogous brain function in humans.

We compared several frequency definitions of spindles: 12–14 Hz^6^ as is most often used in humans, a more lenient range, 9–16 Hz^18^, and based on visual inspection of canine EEG by Pákozdy *et al*., 5–12 Hz^1^. Other commonly used criteria as amplitude and minimal duration were the same under the three different frequency regimes^6,53^.

Only events detected in the 9–16 Hz range displayed a relationship with memory, which was measured in two different ways. There was a rise in spindle density in the learning condition as compared to the control condition. Condition specific effects of learning on spindle activity are also known from work with humans^27,66^. There was furthermore a strong correlation between the density of spindles observed during the learning condition and the rise in correct responses to the newly learned commands, before and after sleep. This type of comparison is also found in the literature^16,67^ and was significant both with and without the adjustment for additional predictors (age, sex). Importantly, neither learning-associated effect was found for detections using lower and higher harmonics of the 9–16 Hz frequency range (see supplement). We can also exclude alpha as an alternative explanation, which in the original study using this data-set declined with learning^4^ and therefore cannot explain the observed findings.

A strong association with sex was found in all definitions with females displaying a higher density of spindles than males. This is also true in humans, where already in very small samples the difference in spindle density can be roughly 2:1 for women versus men^46^. The magnitude of the relationship is similar in our sample and is connected to a difference in learning gain (increase in performance on the newly learned commands), while also particularly pronounced in the learning condition. Excluding neutered animals only increased the observed effect, suggesting that the difference might be due to gonadal hormones. The sex difference, however was not only not specific to any of the tested frequency ranges, but also found in the lower harmonics of the 9–16 Hz range (supplementary). In light of these findings we can only conclude with certainty that sex has an effect on oscillations below 18 Hz. Interestingly, a sex difference was not found if a 7.5–12.5 Hz search range was used (supplementary), which was previously defined as the canine alpha^5^.

Age effects were only found for detections in the 9–16 Hz definition and only when corrected for other predictors (learning gain, sex). In the learning condition the density of spindles was significantly rising with age. While spindles overall decline with age^42,43^, it should be noted that no age effect on learning was revealed in our analyses or reported in the original study using this data set^4^. This could suggest that older dogs need a higher density of spindles to achieve an overall similar performance on the learning task. However, an age-related rise was also found for detections in the higher harmonics of the 9–16 Hz range. This casts doubt on whether this effect can be specifically related to spindle-analogue activity.

9–16 Hz transients were further used to inquire if a division into fast and slow spindles^19^ applies in the dog. To this end the detections were divided in two groups based on the 13 Hz cut-off used by Schabus *et al*.^54^ on human data (see Supplementary Figure S2 for examples from each category). In both groups sex remained a significant predictor of spindle density, but only slow spindles maintained the previously observed relationships with learning and memory. Slow spindles are localized in frontal areas^21^, where associations between spindling and verbal memory have been reported for humans^16^. A specific association of slow spindles with learning of verbal commands squares with other findings suggesting that the canine brain processes aspects of verbal information similar to the human brain^64,68^. Only frequency, amplitude and density of fast spindles, however, did predict age similarly as in the human literature, where aging causes spindle density and amplitude to drop^40^, but frequency to rise^39^. Of these effects only the decline in amplitude was significant in post-hoc testing, but needs to be taken with caution as well, since the correlation might be biased by a single data point (see Supplementary Figure S3). The results concerning fast spindles might be overall less reliable, as for most dogs a single peak in sigma power was found below 13 Hz (see Supplementary Table S2) and generally fewer fast transients were observed per dog and session (see Supplementary Figure S1 for a count of detections per frequency, across dogs and for each session). It is plausible to assume that the more posterior fast spindles were not as easily detected with our anteriorly placed active electrode, and in particular considering the limitations of bipolar derivations. Highly synchronous events are likely to be cancelled out, if the active electrode is placed far from their source. Therefore, we expect a detection bias in favour of slow spindles. Only two dogs displayed a bimodal distribution of sigma power and in each the peaks were below and above the 13 Hz cut-off proposed by Schabus *et al*.^54^.

Detections in the 9–16 Hz range displayed the strongest overall analogy to human spindles. The result is not entirely surprising. In the human literature, the narrow 12–14 Hz definition is seldom used in automated sleep spindle detection techniques^21^. Nonclercq *et al*.^53^ used it in developing their method, but the validation was done with visual inspection only. Frequency ranges specifically proposed for dogs were also based on visual inspection alone^1,59^, whereas in humans, studies that describe relationships to cognition or memory commonly operate with a broader frequency range like 9–16 Hz^18,69^ or 11–16 Hz^15,16^. Although for many mammalian species spindling has been claimed of much lower frequency than in humans^8,9^, studies in rodents which explored a relationship between sleep spindles and learning could not confirm this^29,30,70–72^.

Part of this discrepancy might be explained by the notion that, even for experts, during visual scoring a portion of spindles will be effectively hidden by other activity. Some of the more elaborate automated search algorithms indeed operate on this assumption^13,14^.

It can be concluded that sleep spindles in the dog are quantifiable with the previously established polysomnography method^3^ and the criteria proposed by Nonclercq *et al*.^53^, albeit with some minor alterations like pre-filtering parameters and amplitude calculation which were adapted to the requirements of our data. These EEG transients are not only similar to human spindles with regard to the criteria by which they have been selected, but show a comparable, functional relationship to memory and sexual dimorphism. Age effects could mostly be observed in models correcting for other predictors and/or detections analogous of human fast spindles. The latter, however, must in the future be approached with alternative recording arrangements, as the set-up we used appears to more reliably measure the slow spindle variety.

An important implication of this work is that spindling frequency in dogs and humans could be more similar than previous estimates^1,9^ suggest. In addition to what has been observed in rodents^29,30,70–72^, our results add to a growing body of evidence against former claims, placing the frequency range of spindles for many mammalian species far below sigma^9^. Regarding humans and dogs in particular, a more similar frequency also adds to previous arguments for the dog as a model of human aging^65^, as sleep spindles are established biomarkers of healthy and pathological changes in the elderly^39,40,42^. Another application, easier to consolidate in the near future, is to advance the utility of diagnostic EEG in the veterinary field, a technology veterinarians have already started exploring^1^. This study is a first step in this direction and the first to our knowledge to test a relationship between spindles and brain function in the dog.

### Data availability

We agree to provide free access to our data and scripts.

## Electronic supplementary material

Supplementary Results

Data Files and Data Descriptor
