# Author Correction: EEG Transients in the Sigma Range During non-REM Sleep Predict Learning in Dogs

**DOI:** 10.1038/s41598-018-24096-6

**Published:** 2018-04-12

**Authors:** Ivaylo Borislavov Iotchev, Anna Kis, Róbert Bódizs, Gilles van Luijtelaar, Enikő Kubinyi

**Affiliations:** 1 Department of Ethology, Eötvös Loránd University, Budapest, Hungary; 2 Institute of Cognitive Neuroscience and Psychology, Hungarian Academy of Sciences, Budapest, Hungary; 3 Institute of Behavioural Sciences, Semmelweis University, Budapest, Hungary; 4 Donders Centre of Cognition, Radboud University, Nijmegen, The Netherlands

Correction to: *Scientific Reports* 10.1038/s41598-017-13278-3, published online 11 October 2017

This Article contains multiple errors. In the Methods section under subheading ‘Subjects and Behavioural paradigm’.

“15 adult pet dogs, mean age ± SD: 3.67 ± 1.91; 8 males, 7 females; from 7 pure breeds (3 Border Collies, 2 Golden Retrievers, 1 Labrador Retriever, 1 Poodle, 1 Belgian Shepherd, 1 Puli, 1 Miniature Schnauzer) and 3 mixed breeds (3 unknown, 1 mixed Briard and 1 mixed Malinois), participated three times in 3-hour-long polysomnography recordings^3^, on a total of 3 days (see Fig. 1)”.

should read:

“15 adult pet dogs, mean age ± SD: 3.87 ± 2.17; 8 males, 7 females; from 7 pure breeds (3 Border Collies, 2 Golden Retrievers, 1 Labrador Retriever, 1 Poodle, 1 Belgian Shepherd, 1 Puli, 1 Miniature Schnauzer) and 3 mixed breeds (3 unknown, 1 mixed Briard and 1 mixed Malinois), participated three times in 3-hour-long polysomnography recordings^3^, on a total of 3 days (see Fig. 1)”.

In the Results section under subheading ‘Age, sex, and learning gain’.

“An initial exploration into how learning gain (difference in percentage correct responses after sleep – before sleep in the learning condition) was predicted by sex and age revealed no effect of age (GLMM, F_1,12_ = 0.759, P = 0.401), but a significant effect of sex (GLMM, F_1,12_ = 6.948, P = 0.022). Females displayed a higher learning gain (15.6 ± 3.6 versus 4.4 ± 2.2, means ± SE, t_12_ = 2.636, P = 0.022), see Fig. 3B.

Next the overall predictive strength of detections from each frequency-definition was compared by testing how age, sex and learning gain would predict spindle density in the learning condition. Transients in the 5–12 Hz and 12–14 Hz range showed no relationship to learning or age (see supplementary). Below we present the results for transients in the 9–16 Hz range (Fig. 3).

We found that spindle density in the learning condition increased with learning gain (GLMM, F_1,11_ = 7.656, P = 0.018). This relationship remained significant in post-hoc testing (GLMM, F_1,13_ = 9.293, P = 0.009, Fig. 3A). Spindle density also increased with age (GLMM, F_1,11_ = 6.492, P = 0.027) and was different for the sexes (GLMM, F_1,11_ = 14.489, P = 0.003). Females had a higher spindle density than males (4.75 ± 0.2 versus 2.69 ± 0.4, means ± SE, t_11_ = 4.787, P = 0.001, Fig. 3C), but the effect of age was not significant post-hoc (GLMM, F_1,13_ = 0.178, P = 0.68)”.

should read:

“An initial exploration into how learning gain (difference in percentage correct responses after sleep – before sleep in the learning condition) was predicted by sex and age revealed no effect of age (GLMM, F_1,12_ = 0.075, P = 0.789), but a significant effect of sex (GLMM, F_1,12_ = 5.591, P = 0.036). Females displayed a higher learning gain (15.6 ± 3.6 versus 4.4 ± 2.2, means ± SE, t_12_ = 2.636, P = 0.022), see Fig. 3B.

Next the overall predictive strength of detections from each frequency-definition was compared by testing how age, sex and learning gain would predict spindle density in the learning condition. Transients in the 5–12 Hz and 12–14 Hz range showed no relationship to learning or age (see supplementary). Below we present the results for transients in the 9–16 Hz range (Fig. 3).

We found that spindle density in the learning condition increased with learning gain (GLMM, F_1,11_ = 8.798, P = 0.013). This relationship remained significant in post-hoc testing (GLMM, F_1,13_ = 9.293, P = 0.009, Fig. 3A). Spindle density also increased with age (GLMM, F_1,11_ = 7.869, P = 0.017) and was different for the sexes (GLMM, F_1,11_ = 10.956, P = 0.007). Females had a higher spindle density than males (4.75 ± 0.2 versus 2.69 ± 0.4, means ± SE, t_11_ = 4.787, P = 0.001, Fig. 3C), but the effect of age was not significant post-hoc (GLMM, F_1,13_ = 0.178, P = 0.68)”.

In the Results section under subheading ‘Slow and fast spindles’.

“1. Slow spindles: In the learning condition the density of slow spindles was significantly predicted by learning gain (GLMM, F_1,11_ = 10.412, P = 0.008). This effect was also significant post-hoc (GLMM, F_1,13_ = 11.661, P = 0.005, Fig. 6A). Sex was a significant predictor (GLMM, F_1,11_ = 7.364, P = 0.02). Females had more spindles/minute than males (4.1 ± 0.3 versus 2.6 ± 0.4, means ± SE, t_11_ = 3.031, P = 0.011, Fig. 6B). There was a trend for density to increase with age (GLMM, F_1,11_ = 4.124, P = 0.067). There was also a trend for more spindles/minute in the learning condition as compared to the control condition (3.4 ± 0.4 versus 2.6 ± 0.5, means ± SE, t_14_ = 2.135, P = 0.051). This effect was significant upon excluding dogs with more than 10 days waiting time between the EEG sessions (3.2 ± 0.5 versus 2.01 ± 0.5, means ± SE, t_10_ = 2.959, P = 0.014, Fig. 6C). Age was not predicted by the mean amplitude (GLMM, F_1,11_ = 0.285, P = 0.604), mean frequency (GLMM, F_1,11_ = 1.351, P = 0.27) or mean density of slow spindles (GLMM, F_1,11_ = 0.673, P = 0.429).

2. Fast spindles: The density of fast spindles was not predicted by learning gain (GLMM, F_1,9_ = 0.005, P = 0.946) or age (GLMM, F_1,9_ = 0.093, P = 0.768) but was significantly predicted by sex (GLMM, F_1,9_ = 10.83, P = 0.009). Females displayed more fast spindles/minute than males (0.8 ± 0.2 versus 0.2 ± 0.1, means ± SE, t_9_ = 2.631, P = 0.027, Fig. 6D). Condition had no effect on the density of fast spindles (t_14_ = 1.557, P = 0.142). Age was predicted by the mean amplitude of fast spindles (GLMM, F_1,8_ = 27.608, P = 0.001), which decreased with years of age; was predicted by a decrease in their density (GLMM, F_1,8_ = 6.454, P = 0.035), while frequency showed a trend to increase with age (GLMM, F_1,8_ = 4.132, P = 0.077). The effect of density was not significant post-hoc (GLMM, F_1,10_ = 0.607, P = 0.454), neither the effect of frequency (GLMM, F_1,10_ = 0.082, P = 0.781), but the mean amplitude remained a significant predictor of age (1.8 ± 0.1 versus 1.5 ± 0.3, means ± SE for dogs older and younger than 6 years^55^, amplitude measured as standard deviation above baseline; GLMM, F_1,10_ = 12.426, P = 0.005). See Fig. 6 for a summary of these results”.

should read:

“1. Slow spindles: In the learning condition the density of slow spindles was significantly predicted by learning gain (GLMM, F_1,11_ = 10.634, P = 0.008). This effect was also significant post-hoc (GLMM, F_1,13_ = 11.661, P = 0.005, Fig. 6A). Sex was a significant predictor (GLMM, F_1,11_ = 5.419, P = 0.04). Females had more spindles/minute than males (4.1 ± 0.3 versus 2.6 ± 0.4, means ± SE, t_11_ = 3.031, P = 0.011, Fig. 6B). In the learning condition the density of slow spindles was significantly predicted by learning gain (GLMM, F_1,11_ = 10.634, P = 0.008). There was also a trend for more spindles/minute in the learning condition as compared to the control condition (3.4 ± 0.4 versus 2.6 ± 0.5, means ± SE, t_14_ = 2.135, P = 0.051). This effect was significant upon excluding dogs with more than 10 days waiting time between the EEG sessions (3.2 ± 0.5 versus 2.01 ± 0.5, means ± SE, t_10_ = 2.959, P = 0.014, Fig. 6C). Age was not predicted by the mean amplitude (GLMM, F_1,11_ = 0.257, P = 0.622), mean frequency (GLMM, F_1,11_ = 0.268, P = 0.615) or mean density of slow spindles (GLMM, F_1,11_ = 0.003, P = 0.959).

2. Fast spindles: The density of fast spindles was not predicted by learning gain (GLMM, F_1,9_ = 0.001, P = 0.973) or age (GLMM, F_1,9_ = 0.138, P = 0.719) but was significantly predicted by sex (GLMM, F_1,9_ = 11.521, P = 0.008). Females displayed more fast spindles/minute than males (0.8 ± 0.2 versus 0.2 ± 0.1, means ± SE, t_9_ = 2.631, P = 0.027, Fig. 6D). Condition had no effect on the density of fast spindles (t_14_ = 1.557, P = 0.142). Age was predicted by the mean amplitude of fast spindles (GLMM, F_1,8 = _27.651, P = 0.001), which decreased with years of age. There was a trend for their density to decrease as well (GLMM, F_1,8 = _3.516, P = 0.098), while frequency did not predict age (GLMM, F_1,8 = _2.502, P = 0.152). The effect of density was not significant post-hoc (GLMM, F_1,10 = _0.132, P = 0.724), but mean amplitude remained a significant predictor of age (1.7 ± 0.1 versus 1.6 ± 0.2, means ± SE for dogs older and younger than 6 years^55^, amplitude measured as standard deviation above baseline; GLMM, F_1,10_ = 17.454, P = 0.002). The effect of density was not significant post-hoc (GLMM, F_1,10_ = 0.132, P = 0.724), neither the effect of frequency (GLMM, F_1,10_ = 0.082, P = 0.781), but the mean amplitude remained a significant predictor of age (1.7 ± 0.1 versus 1.6 ± 0.2, means ± SE for dogs older and younger than 6 years, amplitude measured as standard deviation above baseline; GLMM, F_1,10_ = 17.454, P = 0.002). See Fig. 6 for a summary of these results”.

In the discussion section:

“In both groups sex remained a significant predictor of spindle density, but only slow spindles maintained the previously observed relationships with learning and memory. Slow spindles are localized in frontal areas^21^, where associations between spindling and verbal memory have been reported for humans^16^. A specific association of slow spindles with learning of verbal commands squares with other findings suggesting that the canine brain processes aspects of verbal information similar to the human brain^64, 68^. Only frequency, amplitude and density of fast spindles, however, did predict age similarly as in the human literature, where aging causes spindle density and amplitude to drop^40^, but frequency to rise^39^”.

should read:

“In both groups sex remained a significant predictor of spindle density, but only slow spindles maintained the previously observed relationships with learning and memory and age. Slow spindles are localized in frontal areas^21^, where associations between spindling and verbal memory have been reported for humans^16^. A specific association of slow spindles with learning of verbal commands squares with other findings suggesting that the canine brain processes aspects of verbal information similar to the human brain^64, 68^. Only the amplitude and density of fast spindles, however, did predict age similarly as in the human literature, where aging causes spindle density and amplitude to drop^40^, but frequency to rise^39^. The decrease in density was merely a trend and only the decline in amplitude was significant in post-hoc testing, but needs to be taken with caution as well, since the correlation might be biased by a single data point (see supplementary Figure S3)”.

In addition, this Article contains an error in the Figure legends throughout the paper. All instances of “medians and standard errors” should be “means ± SD.”

