# Supplementary material for: EEG Transients in the Sigma Range During non-REM Sleep Predict Learning in Dogs
Source: Sci Rep. 2017 Oct 11;7:12936. doi: 10.1038/s41598-017-13278-3 (PMC5636833; doi:10.1038/s41598-017-13278-3)
Supplement: Supplementary file 1 — Supplementary Results [file 41598_2017_13278_MOESM1_ESM.pdf]

Supplementary

EEG Transients in the Sigma Range During non-REM Sleep Predict Learning in Dogs

Ivaylo Borislavov Iotchev[a], Anna Kis[b], Róbert Bódizs[c, d], Gilles van Luijtelaar[e], Enikő Kubinyi[a]

[a] Department of Ethology, Eötvös Loránd University, Budapest, Hungary

[b] Institute of Cognitive Neuroscience and Psychology, Hungarian Academy of Sciences, Budapest, Hungary

[c] Institute of Behavioural Sciences, Semmelweis University, Budapest, Hungary

[d] Department of General Psychology, Pázmány Péter Catholic University, Budapest, Hungary

[e] Donders Centre of Cognition, Radboud University, Nijmegen, The Netherlands

Absolute performance on the learning task

Dog	correct trials - control	correct trials - test	correct trials - retest
Álmos	16	11	12
Csinszka	18	9	12
Dorisz	16	12	12
Füles	14	12	11
Glenn	15	10	15
Grog	14	12	13
Kendra	15	9	13
Kenny	18	17	18
Maya	16	11	15
Naty	17	11	13
Onix	12	12	12
Rumli	16	13	13
Smafu	16	10	12
Tódor	17	16	17
Wicca	15	12	15

Table S1. Number of correct trials (from a total of 18) for each dog and session.

Additional results, transients in the 12-14 Hz and 5-12 Hz range

Analysis effects of age, sex and learning on spindle density in the learning condition. Below the results for the 12-14 Hz and 5-12 Hz transients:

1. 12-14 Hz transients: The density of detections (spindles/minute) in the learning condition was not affected by learning gain (GLMM, $F_{1,10}$ = 1.109, P = 0.317) or age (GLMM, $F_{1,10}$ = 2.165, P = 0.172), but was predicted by sex (GLMM, $F_{1,10}$ = 26.111, P < 0.001). Females displayed more spindles per minute than males (0.99 ± 0.1 versus 0.36 ± 0.06, means ± SE, t_{10} = 4.834, P = 0.001).

2. 5-12 Hz transients: There was no effect of learning gain (GLMM, $F_{1,11}$ = 0.181, P = 0.678) or age (GLMM, $F_{1,11}$ = 0.652, P = 0.436), but again the effect of sex was significant (GLMM, $F_{1,11}$ = 7.327, P = 0.02) with females exhibiting more spindles/minute than males (12.67 ± 0.5 versus 7.61 ± 1.4, means ± SE, t_{11} = 3.376, P = 0.006).

Local spectral peaks in the dogs' alleged spindle-frequency

Although an absolute cut-off frequency has been used in previous work on humans, to delineate slow from fast spindles (13 Hz in Schabus et al.[1]), we further inquired if such a cut-off correctly describes the distribution of frequency power in the dogs. To this end we looked for local peaks in the power of each dog's estimated spindle-frequency range, based on the 9-16 Hz search-criterion. The estimated spindle-frequency range (see methods) was the average frequency of the detections +/- 2 standard deviations. Results are shown in Table S2. In addition we present, across animals, a distribution histogram of detections per frequency for each the control and learning condition (Figure S1).

Dog	spectral peaks sigma (control condtion):	spectral peaks sigma (learning condition):
Álmos	8.7 Hz	8 Hz
Csinszka	14.1 Hz	9 Hz
Dorisz	8.7 Hz	9.6 Hz, 13.1 Hz
Füles	12.4 Hz	9.8 Hz
Glenn	8.6 Hz	9.3 Hz
Grog	9.9 Hz	8.9 Hz
Kendra	9.1 Hz	8.7 Hz
Kenny	8.8 Hz	8.6 Hz
Maya	8.8 Hz	8.8 Hz
Naty	9 Hz	9.3 Hz
Onix	12.7 Hz	8.9 Hz
Rumli	9.9 Hz	9.9 Hz
Smafu	8.9 Hz	9.1 Hz, 14.7 Hz
Tódor	8.9 Hz	8.5 Hz
Wicca	7.9 Hz	8.4 Hz

Table S2. Local peaks in the power of the estimated sigma-range of each dog, listed for each dog and session.

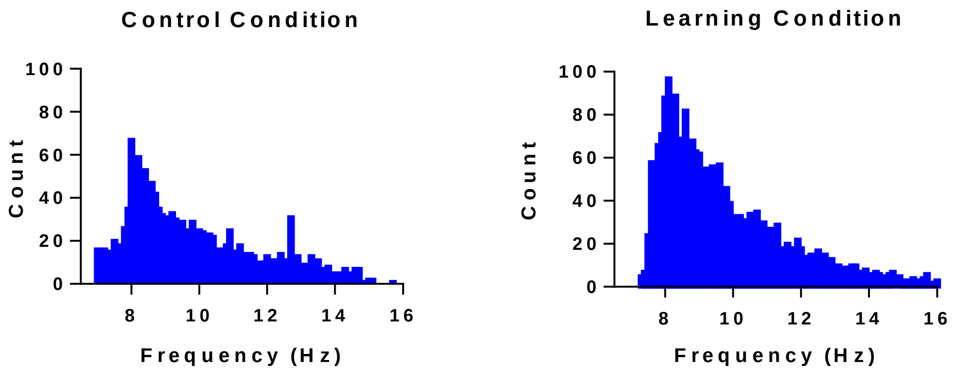

Figure S1. Count of detections, per frequency and across animals, marked as spindle-containing time windows in the 9-16 Hz search for each the control and learning condition.

Examples of spindle events, slow (<= 13 Hz) and fast (>= 13 Hz)

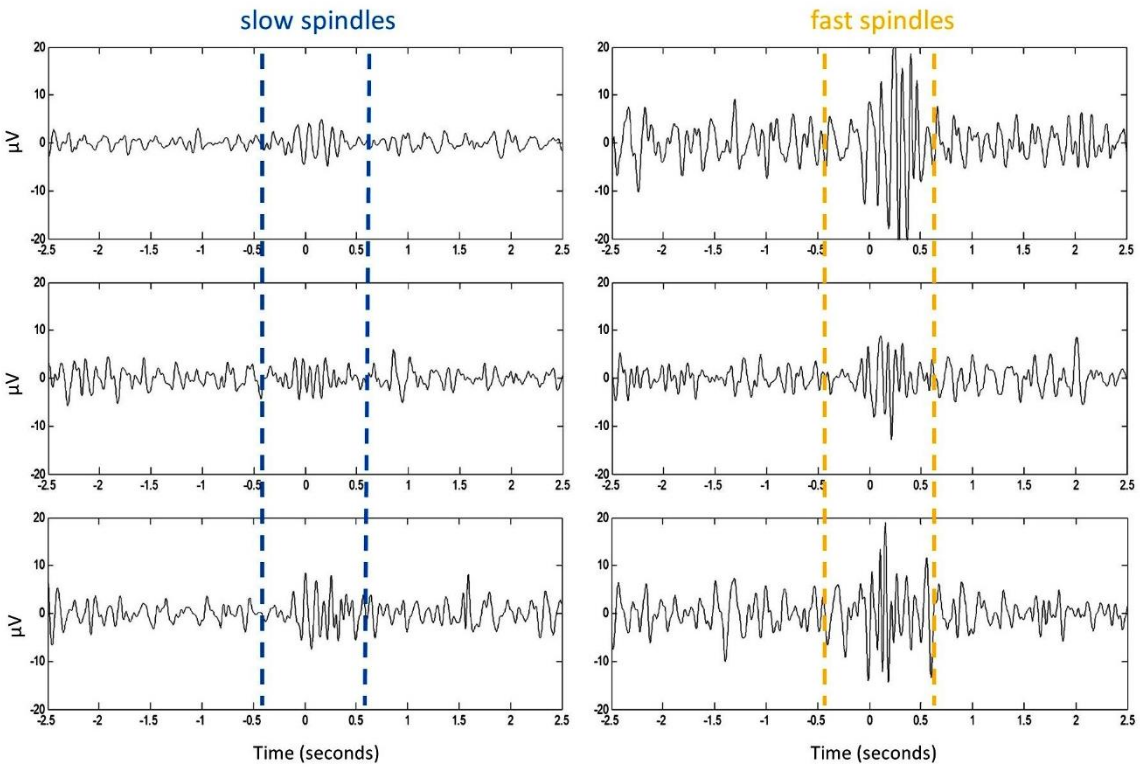

Figure S2. Examples of various detections in the 9-16 Hz range, classified either as 'slow-' or 'fast spindles'. Shown are 2.5 seconds of the filtered signal before and after time point of detection, for amplitude values between 20 and -20 μV.

Fast spindles, mean amplitude as a predictor of age

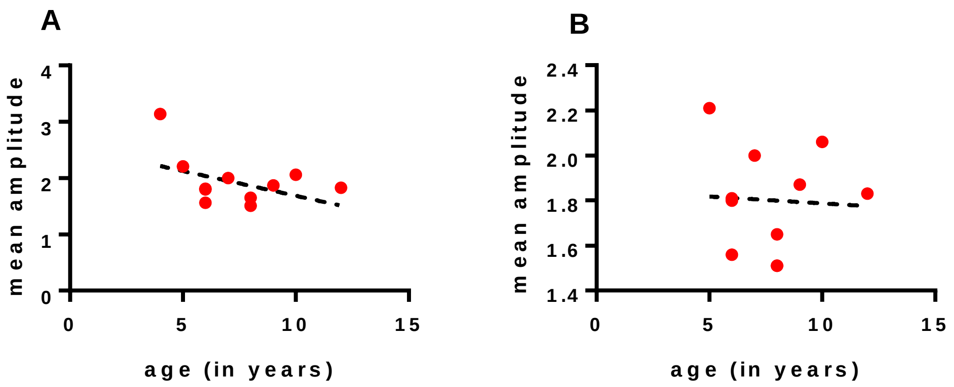

Figure S3. There was an association between age (in years) and the mean amplitude of fast spindles (A), however this effect seemed dependent on a single observation and disappeared when the data point was removed (B). The scaling in (A) and (B) is adjusted to the maximum values.

Control Analyses

To ensure that any effects observed were due to activity in the targeted frequencies and not artefacts caused by their harmonics, we repeated the above comparisons with the first lower and the first higher harmonic of the 9-16 Hz range. We focused on this range, because effects of learning and age were only found for 9-16 Hz transients, whereas sex differences were found in every range. In other words, there were no effects specific to the 5-12 Hz or 12-14 Hz detections. The higher harmonic, 18-32 Hz, was tested with 0.25 seconds long time windows and events were considered separate if they were more than 0.25 seconds apart. The lower harmonic, 4.5-8 Hz, was tested with one-second-long time windows and detections were considered separate events if more than a second apart.

1. In the learning condition, density of detections in the lower harmonics was not predicted by learning gain (GLMM, $F_{1,11}$ = 0.92, P = 0.358) or age (GLMM, $F_{1,11}$ = 0.683, P = 0.426), but there was an effect of sex (GLMM, $F_{1,11}$ = 8.191, P = 0.015). Females displayed a higher density than males (5.7 ± 0.2 versus 3.2 ± 0.6, means ± SE, t_{11} = 3.681, P = 0.004).

2. Density of detections in the higher harmonics, during the learning condition, did not correlate with learning gain (GLMM, $F_{1,6}$ = 1.156, P = 0.324) or sex (GLMM, $F_{1,6}$ = 2.119,

P = 0.196), but increased with age (GLMM, $F_{1,6}$ = 8.374, P = 0.028). The effect of age was marginally significant in post-hoc testing (GLMM, $F_{1,8}$ = 3.67, P = 0.092).

We also repeated the between condition comparisons with each harmonic. There was no difference in density between the learning and control condition for detections in the lower harmonics (t_{14} = 0.674, P = 0.511) or higher harmonics (t_{14} = 0.033, P = 0.974).

To control for alpha activity, we also ran our analyses with the target range 7.5-12.5 Hz, previously used in dogs to measure alpha[2]. We found no effect of learning gain (GLMM, $F_{1,11}$ = 1.089, P = 0.319), age (GLMM, $F_{1,11}$ = 0.038, P = 0.85) or sex (GLMM, $F_{1,11}$ = 0.493, P = 0.497) for the density of detections in the learning condition. There was also no difference between the control and learning condition (t_{14} = 0.958, P = 0.354).

One final control was to exclude animals whose mean amplitude was more than 2 standard deviations above baseline. Although our algorithm restricts the final count to those events with an amplitude within 2 standard deviations of all initial detections, we reasoned that outliers would more easily bypass this control if an animal has overall too few detections in a given session. In the learning condition only one animal's mean amplitude exceeded 2 standard deviations. Excluding this dog, the spindle density was still significantly rising with age (GLMM, $F_{1,10}$ = 7.961, P = 0.018), learning gain (GLMM, $F_{1,10}$ = 8.067, P = 0.018), and was different for the sexes (GLMM, $F_{1,10}$ = 18.469, P = 0.002); Females displayed a higher density than males (4.8 ± 0.2 versus 3 ± 0.3, means ± SE, t = 5.03, P = 0.001). Learning gain remained significant in post-hoc testing (GLMM, $F_{1,12}$ = 9.386, P = 0.01), but age had no effect as a sole predictor (GLMM, $F_{1,12}$ = 0.001, P = 0.983). In the control condition three more dogs were excluded for displaying a mean amplitude exceeding 2 standard deviations. There was still a trend for a higher density in the learning condition (4.3 ± 0.4 versus 3.6 ± 0.4, means ± SE, t_{10} = 1.838, P = 0.096), but this difference was now only significant for dogs with less than 10 days waiting time between the tests (4.4 ± 0.6 versus 3.1 ± 0.6, means ± SE, t_6 = 3.67, P = 0.01).

References

1. Schabus M, Hödlmoser K, Gruber G, et al. Sleep spindle-related activity in the human EEG and its relation to general cognitive and learning abilities. *Eur J Neurosci*. 2006;23(7):1738-1746. doi:10.1111/j.1460-9568.2006.04694.x.

2. Wauquier A, De Ryck M, Van den Broeck W, Van Loon J, Melis W, Janssen P. Relationships between quantitative EEG measures and pharmacodynamics of alfentanil in dogs. *Electroencephalogr Clin Neurophysiol*. 1988;69(6):550-560. doi:10.1016/0013-4694(88)90167-8.
