# Supplementary material for: EEG Transients in the Sigma Range During non-REM Sleep Predict Learning in Dogs
Source: Sci Rep. 2017 Oct 11;7:12936. doi: 10.1038/s41598-017-13278-3 (PMC5636833; doi:10.1038/s41598-017-13278-3)
Supplement: Supplementary file 2 — Data Files and Data Descriptor [file 41598_2017_13278_MOESM2_ESM.zip › Data_Descriptor_SREP-17-22880A.docx]

The following data sets correspond to the following data discussed in the article „EEG Transients in the Sigma Range During non-REM Sleep Predict Learning in Dogs”

spindle_data_good_5_12Hz.sav => transients in the 5-12 Hz search range, SPSS data file

spindle_data_good_9_16Hz.sav => transients in the 9-16 Hz search range, SPSS data file

spindle_data_good_12_14Hz.sav => transients in the 12-14 Hz search range, SPSS data file

spindle_data_good_9_13Hz.sav => transients corresponding to ’slow’ spindles

spindle_data_good_13_16Hz.sav => transients correpsonding to ’fast’ spindles

spindle_data_alphacontrol.sav => transients with the spectral definition of canine alpha

spindle_data_good_9_16Hz_higher_harmonics.sav => data set for the higher harmonics

spindle_data_good_9_16Hz_lower_harmonics.sav => data set for the lower harmonics

spindle_hound_2017.m => code for detecting transients in the 9-16 Hz range, variations

differing only in the target frequency were used for transients

with a different frequency definition. Now (Resubmission) we

actually provide all scripts, the naming should be intuitive:

spindle_hound_2017_(**insert target range**).

The variables in the .sav files are all named following the same system:

**control_**... = variables obtained from the control condition

**learn_**... = variables obtained from the learning condition

**baseline_**... = variables obtained from the adaptation condition

**mean_**... = average value across conditions

**Diff_**... = **learn_**... – **control_**...

...**_nr** = absolute count of spindle detections for a dog and condition (e.g. **learn_nr** = detections in the learning condition)

...**_dens** = density i.e. spindles per minute non-REM sleep

...**_freq** = average frequency of the detections for a dog and condition

...**_amp** = amplitude (measured in standard deviations (relative to baseline))

**control_durSWS2** = length of non-REM sleep (in minutes) for the control condition

**learn_dur_SWS2** = length of non-REM sleep (in minutes) for the learning condition

**baseline_SWS2** = length of non-REM sleep (in minutes) for the adaptation condition

**beforesleep** = performance on the task (command learning), during the learning condition, before sleeping

**aftersleep** = performance on the task (command learning), during the learning condition, after sleeping

**control** = performance on the task (command learning) during the control condition

**learninggain** = **aftersleep** – **beforesleep**

**age** = age in years

**sex** = female (0), male (1)

**breed** = name of the breeds

**neutered** = neutered (1), intact (0)

**daysbetween** = how many days passed between the two experimental conditions?

**order_condition** = control first (1), learning first (2)
